# Induced pluripotent stem cells from patients with human fibrodysplasia ossificans progressiva show increased mineralization and cartilage formation

**DOI:** 10.1186/1750-1172-8-190

**Published:** 2013-12-09

**Authors:** Yoshihisa Matsumoto, Yohei Hayashi, Christopher R Schlieve, Makoto Ikeya, Hannah Kim, Trieu D Nguyen, Salma Sami, Shiro Baba, Emilie Barruet, Akira Nasu, Isao Asaka, Takanobu Otsuka, Shinya Yamanaka, Bruce R Conklin, Junya Toguchida, Edward C Hsiao

**Affiliations:** 1 Department of Tissue Regeneration, Institute for Frontier Medical Sciences, Kyoto University, 53 Kawahara-cho, Shogoin, Sakyo-ku 606-8507, Kyoto, Japan; 2 Department of Cell Growth and Differentiation, Center for iPS Cell Research and Application, Kyoto University, 53 Kawahara-cho, Shogoin, Sakyo-ku 606-8507, Kyoto, Japan; 3 Department of Orthopaedic Surgery, Graduate School of Medical Sciences, Nagoya City University, Nagoya 467-8601, Japan; 4 Gladstone Institute of Cardiovascular Disease, San Francisco, CA 94158, USA; 5 Department of Medicine, Duke-NUS Graduate Medical School, Singapore 169857, Singapore; 6 Department of Medicine, Division of Endocrinology and Metabolism and the Institute for Human Genetics, University of California-San Francisco, 513 Parnassus Ave., HSE901G, San Francisco, CA 94143-0794, USA; 7 Department of Orthopaedic Surgery, Graduate School of Medicine, Kyoto University, Kyoto 606-8507, Japan; 8 Department of Fundamental Cell Technology, Center for iPS Cell Research and Application, Kyoto University, Kyoto 606-8507, Japan; 9 Department of Reprogramming Science, Center for iPS Cell Research and Application, Kyoto University, Kyoto 606-8507, Japan; 10 Department of Medicine and Cellular and Molecular Pharmacology, University of California-San Francisco, San Francisco, CA 94143, USA

**Keywords:** Induced pluripotent stem cells, Fibrodysplasia ossificans progressiva, FOP, Disease model in a dish, Endochondral ossification

## Abstract

**Background:**

Abnormal activation of endochondral bone formation in soft tissues causes significant medical diseases associated with disability and pain. Hyperactive mutations in the bone morphogenetic protein (BMP) type 1 receptor ACVR1 lead to fibrodysplasia ossificans progressiva (FOP), a rare genetic disorder characterized by progressive ossification in soft tissues. However, the specific cellular mechanisms are unclear. In addition, the difficulty obtaining tissue samples from FOP patients and the limitations in mouse models of FOP hamper our ability to dissect the pathogenesis of FOP.

**Methods:**

To address these challenges and develop a “disease model in a dish”, we created human induced pluripotent stem cells (iPS cells) derived from normal and FOP dermal fibroblasts by two separate methods, retroviral integration or integration-free episomal vectors. We tested if the ability to contribute to different steps of endochondral bone formation was different in FOP *vs.* control iPS cells.

**Results:**

Remarkably, FOP iPS cells showed increased mineralization and enhanced chondrogenesis *in vitro*. The mineralization phenotypes could be suppressed with a small-molecule inhibitor of BMP signaling, DMH1. Our results indicate that the FOP ACVR1 R206H mutation favors chondrogenesis and increases mineral deposition *in vitro*.

**Conclusions:**

Our findings establish a FOP disease cell model for *in vitro* experimentation and provide a proof-of-concept for using human iPS cell models to understand human skeletal disorders.

## Background

Tissue mineralization is one of the most dramatic transitions in normal development and disease. Hard tissues, such as bone and teeth, maintain body structure, protect vital organs, and facilitate eating. Abnormal activation of endochondral bone formation in soft tissues from congenital disorders, such as fibrodysplasia ossificans progressiva (FOP; OMIM #135100) or from trauma or central nervous system injury [1] cause severe disability and pain. Although bone morphogenetic proteins (BMPs) are key regulators of osteogenesis [2] and are implicated in pathologic calcification [3], their specific functions in humans are not well understood. Human induced pluripotent stem cells (iPS cells) derived from patients with genetic mutations affecting key signaling pathways provide a unique opportunity to dissect human development and disease pathogenesis in models amenable to experimental manipulation [4].

FOP is a rare but debilitating disease of heterotopic bone formation associated with mutations in the Activin A Type I receptor (ACVR1) [5], which is activated by BMPs. A single amino acid change (R206H) commonly resulting from a single base mutation (617G > A) is found in the majority of FOP patients and can increase sensitivity of the receptor to BMP ligands [6]. Patients are born without heterotopic ossification yet characteristically form large amounts of bone either spontaneously or after trauma [7]. The heterotopic ossification follows an endochondral bone formation pathway that starts with a pre-cartilaginous fibro-proliferative anlage and eventually mineralizes. The ferocity of these episodes makes it nearly impossible to obtain tissue samples prospectively and significantly complicates surgical management.

Here, we created a series of iPS cells from dermal fibroblasts of donors with FOP by either DNA-integrating retroviral or integration-free episomal plasmid methods [4,8]. The FOP iPS cells showed increased mineral deposition and chondrogenesis, potentially reflecting a predisposition towards endochondral bone formation in FOP. Our findings provide a valuable foundation for human stem cell-based systems to delineate the mechanisms of normal and pathologic skeletal formation.

## Materials and methods

### Cell culture

Primary human dermal fibroblasts (HDFs) from commercial sources or from 3-mm skin biopsies carefully obtained from donors (without causing deep tissue injury) or from surgical excess were cultured [9] and are described in Table 1 and Additional file 1: Table S1. HDFs less than five passages old were used for iPS cell reprogramming. Presence or absence of the *ACVR1* mutation was sequenced and verified as described [5]. Primary human mesenchymal stem cells (hMSCs) were prepared from iliac bone as described previously [10] and expanded as a monolayer.

**Table 1:** Cell lines used in this study

**Name**	**Origin**
**Human dermal fibroblasts**	
HDF-WTa	Skin
HDF-WTb	Skin
FF-WT-BJ	Foreskin (Stemgent 08–0027)
HDF-WT-1323	Fibroblast (Cell Applications, 1323)
HDF-WT-1388	Fibroblast (Cell Applications, 1388)
HDF-WT-TIG120	Collection of Research Bioresources, Japan
HDF-FOP1	Skin Fibroblast, Corielle GM00513
HDF-FOP2	Skin Fibroblast, Corielle GM00783
HDF-FOP3	Skin
HDF-FOP4	Skin
HDF-FOP5	Skin
Control retroviral iPS cell lines (wildtype ACVR1)*	
vWTa	HDF
vWTb	HDF
vWT-TIG120-4f1	HDF
vWT-201B2	HDF
vWT-201B7	HDF
FOP retroviral iPS cell lines (ACVR1 R206H)*	
vFOP1-1	HDF-FOP1
vFOP1-4	HDF-FOP1
vFOP2-1	HDF-FOP2
vFOP2-2	HDF-FOP2
vFOP4-1	HDF-FOP4
vFOP4-3	HDF-FOP4
vFOP5-22	HDF-FOP5
Control episomal iPS cell lines (Wildtype ACVR1)**	
eWT-1323-2	HDF-1323
eWT-1323-4	HDF-1323
eWT-BJ2	FF-BJ
eWT-BJ4	FF-BJ
FOP episomal iPS cell lines (ACVR1 R206H)**	
eFOP1-1	HDF-FOP1
eFOP1-10	HDF-FOP1
eFOP2-3	HDF-FOP2
eFOP2-8	HDF-FOP2
eFOP3-2	HDF-FOP3
eFOP3-4	HDF-FOP3

Summary of the FOP and control human dermal fibroblasts and iPS cell lines used in this study, including naming, classification (control vs. FOP), and original tissue source. HDF, human dermal fibroblast; FF, foreskin fibroblast. Please see Additional file 1: Table S1 for a detailed summary of the iPS cell characterization. Method of making iPS cells: *, Retroviral OSKM ; **, Epi(Y4).

Retroviral [4] and episomal integration-free [8] iPS cells were derived as described. H9 human embryonic stem (ES) cells were from WiCell Research Institute (Madison, WI). All pluripotent cell lines were maintained in mTeSR1 medium (StemCell Technologies, Vancouver, Canada) on growth-factor-reduced Matrigel (BD Biosciences)-coated plates (150–300 μg/ml, 30 min coating) or in primate ES cell medium (ReproCELL, Tokyo, Japan) on mitomycin C-treated or irradiated SNL feeder cells [11]. SNLs were carefully removed by at least one passage in feeder-free conditions before use in differentiation assays. The ROCK inhibitor Y-27632 (10 μM, Tocris Bioscience, Minneapolis, MN) dissolved in DMSO was added to mTeSR1 at passaging and removed the following day. Karyotyping was done by Cell Line Genetics (Madison, WI) or Nihon Gene Research Laboratories (Sendai, Japan). Cells exposed to recombinant BMP4 protein (R&D Systems, Minneapolis, MN) were treated for 45 minutes.

All human tissue collection, human stem cell studies, procedures, and written consents were approved by the UCSF Committee on Human Research, the UCSF Gamete and Embryonic Stem Cell Research Committee, or by the Ethics Committee of the Department of Medicine and Graduate School of Medicine, Kyoto University.

### Embryoid body formation

Embryoid bodies (EBs) were formed from iPS cells or human ES cells once their cultures reached 80% confluence. After washing with PBS, Accutase (StemCell Technologies, Vancouver, Canada) was applied for two minutes to remove cells from the plate. Cells were centrifuged at 175 × g for two minutes and then resuspended in a 4:1 mix of EB differentiation medium (80% Knockout DMEM, 20% FBS, 1% Glutamax, 1% non-essential amino acids, and 0.1 mM 2-mercaptoethanol) and mTeSR1, and supplemented with 10 μM Y-27632. Cells were plated onto ultra-low attachment plates without medium changes for seven days. On day eight, EBs were collected and allowed to settle in a conical tube for 30 minutes. The mixed medium was removed and replaced with 100% EB differentiation medium (Knockout DMEM supplemented with 20% FBS, 1% Glutamax, 1% non-essential amino acids, and 0.1 mM 2-mercaptoethanol) changed every three to four days. EBs were then transferred to gelatinized plates and cultured until day 15 for RNA collection in Trizol (Invitrogen).

### Teratoma formation

iPS cells grown in six-well Matrigel-coated plates to 100% confluence were released with Accutase for 30 secs, rinsed twice with PBS, and resuspended in mTeSR1 supplemented with 10 μM Y-27632. Cells (1 × 10^6^ in 20 μl) were injected into 8–14 week-old male CB17 SCID mice (Charles River) under the testis capsule as described [4]. A minimum of six testes were injected per iPS cell line. Tumors were collected 8–12 weeks after injection, fixed with 10% neutral buffered formalin for 24 hours, and processed for paraffin-embedded sections the Gladstone Histology and Microscopy Core or at the Division of Technical Support of the Institute for Frontier Medical Sciences in Kyoto University. All mouse studies were approved by the UCSF Institutional Animal Care and Use Committee, or performed in strict accordance with the Regulations on Animal Experimentation at Kyoto University.

### Mineralization assay

Primary human MSCs were cultured in OB mineralization medium (DMEM with 20% FBS, glycerol-2-phosphate, 4 μM dexamethasone, 0.1 mM 2-mercaptoethanol, and 50 μg/ml L-ascorbic acid 2-phosphate sesquimagnesium salt hydrate [12]). We could detect mineralization activity after 12 days as increased black staining by von Kossa (Additional file 2: Figure S1B), which was the preferred staining method used because the black mineralization nodules could be easily distinguished from the expected light golden-yellow staining of the cell layer. Human iPS cells maintained in feeder-free conditions were plated in 20% mTeSR1 mixed with 80% OB mineralization medium and Y-27632 (10 μM) at 2 million, 400,000, or 37,500 cells per well of gelatin-coated 6-, 24-, or 96-well plates, respectively. Medium was replaced on day two with 100% OB mineralization medium and changed every other day. Samples for von Kossa staining were fixed in 4% paraformaldehyde (PFA), stained in 5% silver nitrate for 15 minutes, and developed in 5% sodium carbonate/9.25% formaldehyde for two minutes. Regions of increased mineralization (dark black staining) can be seen at the edges of the well, where the culture surface meets the well wall. This was observed for all cell types and was excluded from staining intensity analysis. ImageJ (ver. 1.44o) was used for staining intensity analysis [13]. DMH1 (4-[6-[4-(1-Methylethoxy)phenyl]pyrazolo[1,5-*a*]pyrimidin-3-yl]-quinoline; from Dainippon-Sumitomo Pharma or Nacalai Tesque, Tokyo, Japan) was used at indicated concentrations; levels above 10 μM caused cell death. Scanning electron microscopy was performed on an Olympus TM3000 scanning electron microscope by the Gladstone Microscopy Core.

### Chondrogenic differentiation

Pellet chondrogenic cultures were performed as described [14]. Briefly, human iPS cells collected with a scraper were suspended as clumps in EB formation medium (DMEM, 10% Knockout Serum Replacement (KSR, Invitrogen), 10% FBS) and cultured for seven days on non-adherent bacterial petri dishes. The medium was changed every three days. EBs were landed onto 10-cm gelatin-coated tissue-culture dishes, grown to confluence, released with 0.25% trypsin/EDTA, filtered through a 70-μm cell strainer, and seeded onto new gelatin-coated dishes. Once confluent, cells were collected and 2.5 × 10^5^ cells were placed into a 15-ml polypropylene tube, centrifuged at 1200 rpm for three min, and re-suspended in chondrogenic medium (high-glucose DMEM, with human 10 ng/ml TGF-β3, 100 nM dexamethasone, 6 μg/ml insulin, 100 μM ascorbic acid 2-phosphate, 1 mM sodium pyruvate, 6 μg/ml transferrin, 0.35 mM proline, and 1.25 mg/ml bovine serum albumin). Cells were re-centrifuged and maintained as a small pellet in the conical tubes for 28 days with medium changes every three days. Glycosaminoglycan content was quantified with BLYSCAN Dye and Dissociation reagents (BIOCOLOR, Belfast, UK). DNA content was quantified using a PicoGreen dsDNA quantitation kit (Invitrogen).

### RT-PCR and quantitative PCR expression analysis

Semi-quantitative reverse transcription expression analysis of endogenous pluripotency genes, persistent expression of the retroviral iPS cell induction factors, episomal factors, or episomal gene expression, were performed on DNase-treated RNAs with the primers and PCR conditions as described [4,8]. Quantitative PCR reactions were performed using 10 ng of reverse-transcribed cDNA in a final volume of 5 μl with ABI’s TaqMan Universal PCR Master Mix (Life Technologies) or ABI’s Syber Green PCR Master Mix at the recommended reagent ratios with the primers or probesets listed in Additional file 3: Table S2. Each reaction was run in technical triplicates on an Applied Biosystems 7900HT or Viia7 thermocycler (Life Technologies) and normalized to GAPDH as an endogenous control using protocols recommended by ABI.

### Immunocytochemistry

Cells or EBs were fixed with 4% PFA/PBS for 10 min at room temperature, permeabilized with PBS/0.1% Triton X-100 for 10 min at room temperature, then blocked with 1% BSA. Representative lineages were detected with the following primary antibodies: SSEA3 (0.5 μg/ml, eBiosciences), TRA-1-60 (0.5 μg/ml, eBiosciences), TRA-1-81 (0.5 μg/ml, eBiosciences), NANOG (2 μg/ml, AF1997, R&D Systems), βIII-TUBULIN (1:100, MAB1637, Millipore), glial fibrillary acidic protein (1:500, Z0334, DAKO), α-smooth muscle actin (pre-diluted, N1584, DAKO), α-fetoprotein (2 μg/ml, MAB1368, R&D Systems), and tyrosine hydroxylase (1:100, AB152, Chemicon). Secondary antibodies were obtained from Invitrogen: Alexa488 or 555-conjugated goat anti-mouse IgG (1:200), Alexa488 or 555-conjugated goat anti-rabbit IgG (1:200), and Alexa488 or 555-conjugated donkey anti-goat IgG (1:200). Nuclei were stained with DAPI in the Vectashield set (Vector Laboratories).

### Statistical analysis

Because each iPS cell line was derived clonally, and thus may display different behaviors, we treated each cell line as an individual biological replicate and pooled our results into controls or FOP categories. p values were calculated using the Student’s t-test or Dunnett multiple comparisons t-test. p values ≤ 0.05 were considered statistically significant.

## Results

### ACVR1 R206H mutation is not sufficient to induce mineralization of fibroblasts

FOP patients show dramatic heterotopic bone formation in their soft tissues, yet do not ossify their skin. We asked if the ACVR1 R206H mutation [6] could induce mineralization in primary cells that do not normally form bone. Human dermal fibroblasts (HDFs) from control donors without known skeletal disease and FOP donors were cultured, genotyped (Additional file 2: Figure S1A), and propagated in HDF maintenance medium. For our mineralization assays, we used a culture medium that did not contain any exogenous BMPs that might mask the effects of the ACVR1 R206H mutation. This medium still promoted mineralization in primary human MSCs (Additional file 2: Figure S1B) when compared with standard non-mineralizing MSC maintenance medium. However, FOP HDFs showed no differences in mineralization activity when compared to HDFs cultured in non-mineralizing HDF maintenance medium, even with an additional 6 days of culture (Additional file 2: Figure S1C) and despite detectable increases in BMP-related phospho-SMAD levels in FOP HDFs (data not shown). Thus, the ACVR1 R206H mutation was not sufficient to induce spontaneous mineralization in HDFs *in vitro*.

### Human iPS cells with the ACVR1 mutation are pluripotent

Isolating primary tissues from FOP patients is extremely difficult since injuries, including surgical procedures, induce bone formation flares. iPS cells derived from a small skin biopsy or excess surgical material provide a potential way to create a continuous supply of diseased tissues *in vitro* for experimentation. Since BMPs induce human ES cell differentiation [15], activated BMP signaling by the R206H ACVR1 mutation could adversely affect our ability to create FOP iPS cells. Fortunately our standard iPS cell culture media (mTeSR1) is free of BMPs, thus minimizing the potential activation of the hypersensitive R206H ACVR1 receptor by low levels of BMP.

**Figure 1:**
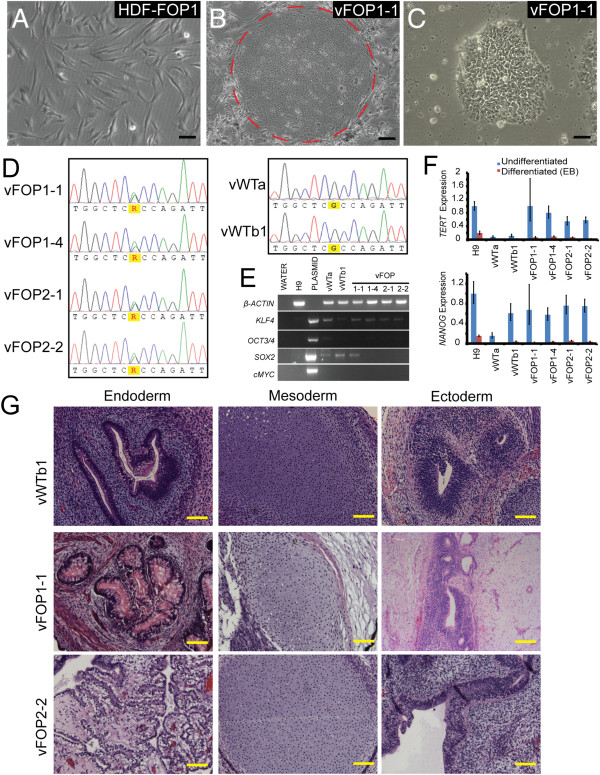
**Characterization of retroviral iPS cell lines. (A)** Representative photos of FOP fibroblasts. **(B)** Subsequent retroviral iPS cell colonies on mouse feeder cells (colony indicated by dashed circle). **(C)** Retroviral iPS cell colonies after transition to feeder-free conditions. Black scale bar = 50 μm. **(D)** Retroviral iPS cell lines are heterozygous for the *ACVR1* R206H (617 G > A) mutation. Control lines vWTa and vWTb1 showed a wildtype *ACVR1* gene. **(E)** The *OCT3/4* and *C-MYC* retroviral transgenes are silenced in the human iPS cells, but *KLF4* and *SOX2* show detectable levels of expression in some lines. **(F)** Pluripotent iPS cells show higher expression of pluripotency markers *TERT* and *NANOG* as compared to differentiated cultures (15-day-old EBs). Gene expression is normalized to *GAPDH* and relative to H9 differentiated EB levels. Error bars are average expression +/− 1 SD of technical triplicates. Gene expression studies were repeated in triplicate. **(G)** Teratoma images showing representative tissues from all three germ layers. Yellow scale bar = 200 μm.

We first created iPS cell lines from banked FOP skin fibroblasts (FOP1 and FOP2). Retroviruses with *OCT4*, *SOX2*, *KLF4*, and *C-MYC*[4] were transfected into two control and two FOP fibroblast lines. We found a significant number of alkaline phosphatase-positive ES cell-like colonies from the FOP fibroblasts. Two control iPS cell lines (vWTa and vWTb) and four FOP iPS lines (vFOP1-1, vFOP1-4, vFOP2-1, and vFOP2-2) were characterized in detail and showed the expected genotypes (Figure 1A-D). All of the retroviral iPS cell lines suppressed the *C-MYC* and *OCT3/4* transgenes (Figure 1E). Although low levels of exogenous *KLF4* and *SOX2* were detected in some lines, the iPS cell lines still expressed genetic markers of pluripotency (Figure 1F) and could form all three germ layers in teratomas (Figure 1G, Additional file 4: Figure S2A) and in EBs (Additional file 4: Figure S2B). While large amounts of cartilage were evident in several of the teratomas derived from FOP iPS cells, comparing the skeletal elements was not possible because of the heterogeneity of the internal structures, overall sizes, and maturity of each individual tumor, even from those derived from the same iPS cell line. All lines retained normal karyotypes (data not shown).

### Retroviral FOP iPS cells show a trend to increased mineralization

**Figure 2:**
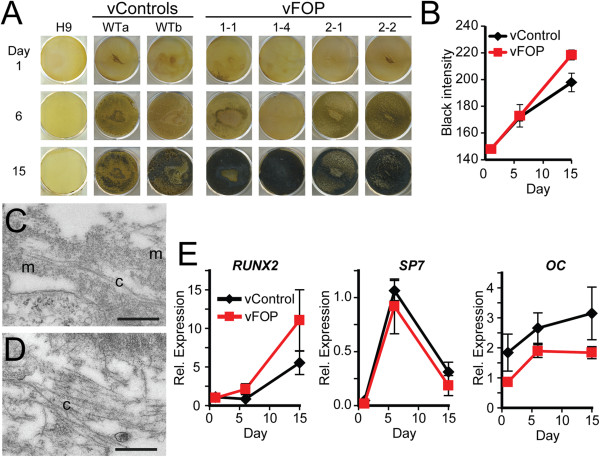
**Increased mineralization in retroviral FOP iPS cells. (A)** von Kossa staining of retroviral control and vFOP1 and vFOP2 iPS cells after culture in mineralizing conditions. Mineral is detected as black staining by von Kossa. **(B)** Quantitation of mineralization intensity. Error bars represent the average of the two control or four FOP iPS lines, with at least three replicates at each time, +/− 1 SEM. **(C, D)** Electron microscopy of day 24 mineralization cultures from vWTb or vFOP1-4 iPS cells showing collagen-like fibers (c) embedded within the mineralizing matrix (m). Scale bar = 500 nm. **(E)** Quantitative PCR expression analysis of the mineralizing cultures. Two control and three FOP lines were pooled together for the expression analysis from triplicates for each time point. Error bars represent average expression +/− 1 SEM.

Since FOP patients develop heterotopic endochondral bone formation, we tested if FOP and control iPS cells might have different predisposition to tissue mineralization *in vitro*. Retroviral FOP iPS cells cultured in mineralizing conditions showed a modest trend (p = 0.15 at 15 days) towards increased mineralization as assessed by von Kossa staining (Figure 2A-B). Using electron microscopy, we confirmed the presence of collagen-like fibers embedded in the mineral (Figure 2C-D), suggesting that the increased mineralization detected by von Kossa was not due to simple calcium/phosphate precipitation that can occur in cultured cell models [16]. qPCR of the mineralizing iPS cells showed minimal changes in the expression of *RUNX2*, *OSX*, and *OC* in FOP and control iPS cells (Figure 2E), consistent with the relatively mild increase in mineralization. Thus, the vFOP iPS cells showed a trend towards increased mineral deposition by histology staining but surprisingly mild changes in gene expression patterns.

**Figure 3:**
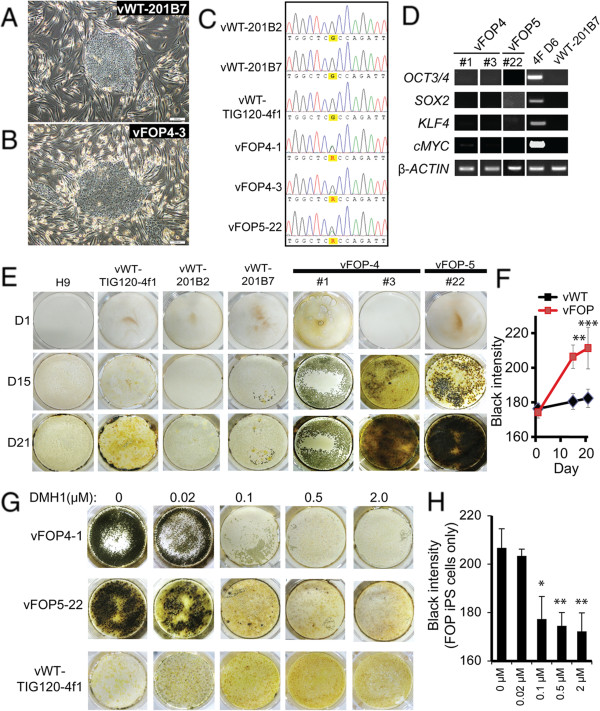
**Increased mineralization in a second cohort of retroviral FOP iPS cells. (A, B)** Representative photos of control and vFOP4 iPS cell colonies on feeders after retroviral iPS cell generation. White scale bar = 200 μm. **(C)** Retroviral iPS cell lines vFOP4-1, vFOP4-3, and vFOP5-22 show the *ACVR1* R206H (617G > A) mutation. **(D)** Retroviral transgene expression in vFOP4 and vFOP5 iPS cell lines by RT-PCR. **(E)** vFOP4, vFOP5, and control iPS cells grown in mineralizing culture conditions. Mineral is detected as black staining with von Kossa. Representative images from triplicate assays with similar results. **(F)** Quantitation of mineralization intensity. Error bars represent the average of the three control cell lines (H9, vWT-TIG120-4f1, and vWT-201B7) or three vFOP iPS lines (vFOP4-1, vFOP4-3, and vFOP5-22), +/− 1 SD. n ≥ 6 measurements from per condition at each timepoint. **, p < 0.01; ***, p < 0.001 by Student’s t-test. **(G)** Increased mineralization by vFOP4 and vFOP5 iPS cells after 15 days of culture can be blocked by treatment with the BMP inhibitor DMH1. Representative experiment from triplicate assays with similar results. **(H)** Quantitation of mineralization intensity after DMH1 treatment of FOP iPS cell lines. n = 3 measurements for each DMH1 concentration and timepoint. *, p < 0.05; **, p < 0.01 by Dunnett’s multiple comparisons t-test compared to the no DMH1 treatment group.

Persistent expression of exogenous transgenes in iPS cells has been reported and may be associated with partial reprogramming [17]. To test if the ACVR1 R206H mutation favored iPS cells that retained expression of the inducing transgenes, and thus created partially-reprogrammed cells that were unable to fully show an osteogenic phenotype, we derived a second cohort of retroviral FOP iPS cell lines from fresh HDFs carefully isolated from two additional FOP patients (FOP4 and FOP5; Figure 3A-C). We used the vWT-TIG120-4f1, vWT-201B2, and vWT-201B7 iPS cell lines [4,18] (here, preceded by vWT- for clarity) as controls. In this additional cohort, *OCT3/4*, *SOX2*, and *KLF4* transgene expression was suppressed (Figure 3D), although a very faint band of C*-MYC* could be detected in vFOP4-1 by RT-PCR. However, quantitative PCR for transgene expression showed significant silencing of all transgenes relative to fibroblasts transduced with retrovirus (Additional file 5: Figure S3A). The vFOP4-1, vFOP4-3, and vFOP5-22 lines also formed teratomas containing all three germ layers (Additional file 5: Figure S3B), had normal karyotypes (Additional file 5: Figure S3C), expressed markers of pluripotency (Additional file 5: Figure S3D), and retained the *ACVR1* R206H (617G > A) mutation (Figure 3C). Furthermore, differentiation of the iPS cells in the same mineralizing conditions used previously showed increased mineralization by histology (Figure 3E, F) and a similar gene expression pattern (Additional file 5: Figure S3E) as seen in the first cohort of retroviral iPS cells. Treating vFOP4-1 and vFOP5-22 iPS cells with the BMP signaling inhibitor DMH1 [19,20] during culture could block the mineralization phenotype (Figure 3G, H).

### FOP iPS cells show increased chondrogenesis

**Figure 4:**
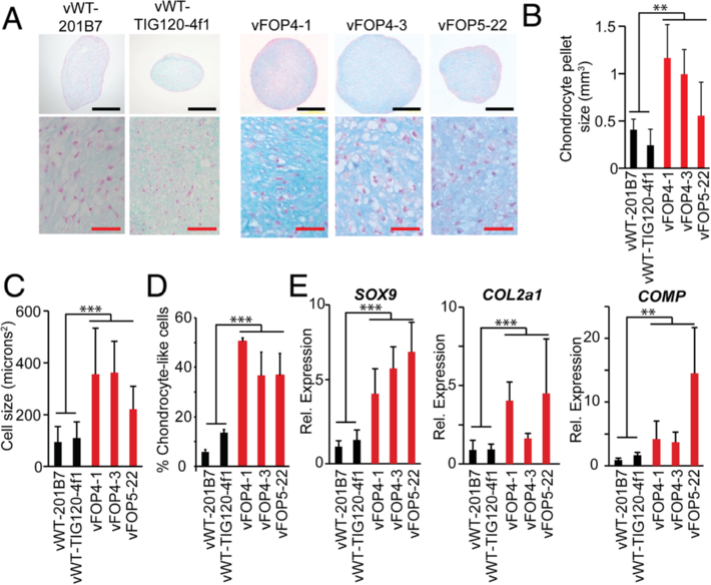
**Increased chondrogenesis in vFOP iPS cells in pellet cultures. (A)** Histology of chondrogenic pellets from vFOP4 and vFOP5 iPS cells show larger chondrocytes and increased proteoglycan. Blue, alcian blue; red, nuclear fast red. Black scale bar = 200 μm; red scale bar = 50 μm. **(B)** Chondrocyte pellet size (calculated volume). Error bars are averages of triplicate experiments, +/− 1 SD. **(C)** Cell size in the chondrogenic pellets. Error bars are averages of triplicate experiments, +/− 1 SD. **(D)** Percentage of chondrocyte-like cells within a culture. Error bars are averages of triplicate experiments, +/− 1 SD. **(E)** Chondrogenic (*SOX9*, *COL2a1*, and *COMP*) gene expression in the pellets. Error bars are averages of triplicate measurements on individual pellets, +/− 1 SD. **, p < 0.01; ***, p < 0.001 by Student’s t-test.

Since the second cohort of retroviral iPS cells showed better retroviral silencing characteristics, we used this set to test if the FOP mutation might affect chondrogenesis in a directed *in vitro* chondrogenesis assay. vFOP iPS cells cultured in 3-dimensional pellet cultures supplemented with TGF-β and dexamethasone [14] produced larger pellets with more ECM-containing chondrocyte-like cells than control iPS cells (Figure 4A, B). Glucose-amino-glycan (GAG) levels were 2–3-fold higher in vFOP iPS cell chondrocyte pellets than in controls (not shown). The vFOP4 and vFOP5 pellets also showed higher numbers of larger chondrocyte-like cells by morphology (Figure 4C-D). Quantitative PCR showed elevated mRNA levels of *SOX9* and *COL2a1* (markers of immature chondrocytes) and *COMP* (marker of mature chondrocytes) in the pellets (Figure 4E).

### Integration-free FOP iPS cells also show increased mineralization

**Figure 5:**
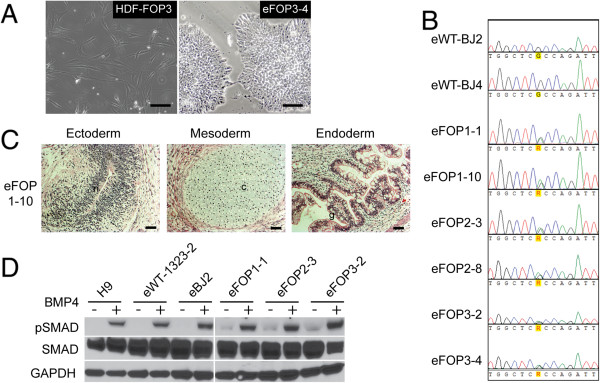
**Integration-free FOP iPS cells. (A)** Representative images of FOP3 dermal fibroblasts subsequently converted to human iPS cell colonies in feeder-free conditions. Black scale bar = 50 μm. **(B)** Human integration-free iPS cells from donors with FOP are heterozygous for the *ACVR1* R206H (617G > A) mutation but control lines are not. **(C)** Representative teratomas from one eFOP iPS cell line showing tissues representing all three germ layers (n, neuronal/primitive neural tube like structure; c, cartilage; g, primitive gut). Additional cell lines are shown in Additional file 6: Figure S4. Black scale bar = 50 μm. **(D)** Western blot showing total SMAD1/5/8 and phospho-SMAD1/5/8 (pSMAD) levels in FOP iPS cell lines relative to control lines with (+) and without (−) 45 minutes of BMP4 exposure. GAPDH, loading control. Representative blot of triplicate experiments.

Since the degree of increased mineralization varied among the different batches of retroviral iPS cells with different levels of transgene silencing (Figures 2A and 3E), we created integration-free iPS cells to test if retroviral transgenes confound our ability to detect the effects of the ACVR1 R206H mutation. We used integration-free episomal vectors to introduce the iPS cell transformation factors *SOX2*, *KLF4*, *OCT4*, *L*-*MYC*, *LIN28*, and *p53* shRNA into control and FOP dermal fibroblasts [8]. We included an expanded collection of control dermal fibroblasts and FOP fibroblasts from skin removed during a medically-necessary surgical procedure (FOP3). We obtained a large number of FOP iPS cell colonies with morphology consistent with human ES cells (Figure 5A). Four lines from two control donors (eWT-1323-2, eWT-1323-4, eWT-BJ2, and eWT-BJ4) and six lines from three FOP donors (eFOP1-1, eFOP1-10, eFOP2-3, eFOP2-8, eFOP3-2, and eFOP3-4) were characterized in detail. All had normal karyotypes (Additional file 6: Figure S4A), retained the R206H ACVR1 mutation (Figure 5B), and expressed pluripotency markers by immunostaining (Additional file 7: Figure S5). Five of the six FOP lines had normal teratoma formation (Figure 5C, Additional file 6: Figure S4B). Line eFOP2-8 lacked clear mature endodermal elements and was excluded from further analyses. Expression analysis suggested eFOP3-4 had low levels of *OCT4* plasmid expression; however, no retained episomal vectors were found by genomic DNA qPCR for the EBNA gene which is located in the episome backbone (Additional file 6: Figure S4C, D). The FOP lines had higher levels of phospho-SMAD 1/5/8 before and after stimulation with BMP4 (Figure 5D), showing responsiveness to exogenous BMP.

**Figure 6:**
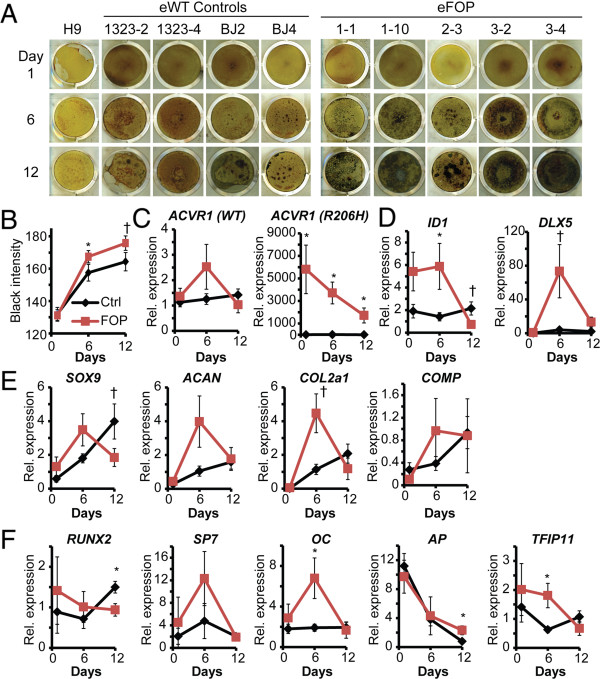
**Increased mineralization capacity in integration-free FOP iPS cells. (A)** eFOP iPS cells show greater mineralization than controls by von Kossa staining. **(B)** Quantitation of mineralization. n = 46–51 wells for each condition, combining at least three replicates of each of the four control iPS lines or five FOP iPS lines. Error bars, +/− 1 SD. *, p < 0.05. †, p < 0.1. **(C)** Quantitative PCR of *ACVR1* (wildtype and R206H mutant) during osteogenic culture. **(D–F)** Quantitative PCR during osteogenic culture of genes downstream of BMP signaling (*ID1* and *DLX5*), chondrogenic (*SOX9*, *ACAN*, *COL2a1,* and *COMP*), osteogenic (*RUNX2*, SP7, and *OC*), and mineralization (*AP* and *TFIP11*) markers. n = 3–6 samples per iPS cell line per condition, each measured in triplicate; Error bars, +/− 1 SEM; *, p < 0.05. †, p < 0.1.

eFOP iPS cells cultured in mineralization medium had significantly increased mineralization activity at day 6 by von Kossa staining (Figure 6A, B), but this difference narrowed by day 12. We found no statistical difference in wildtype *ACVR1* expression even though the R206H *ACVR1* allele expression decreased significantly over the course of the experiment (Figure 6C). *ID1* and *DLX5*, both activated by BMP signaling, were increased at day 6, indicating that the *ACVR1* R206H mutation increased activation of BMP signaling (Figure 6D). Expression analysis showed a trend towards transient increases of chondrogenic markers *COLLAGEN 2* (*COL2a1*) as well as increased expression of the osteogenic marker *OSTEOCALCIN* (*OC*) at day 6 (Figure 6E, F). The FOP iPS cells showed no significant increase in expression of early markers of chondrogenesis (*SOX9*) or osteogenesis (*RUNX2)* at the time points we assayed; in fact, levels of these early markers were higher in control iPS cells as compared to the FOP iPS cells (Figure 6E, F). FOP iPS cells also showed increased expression of genes associated with mineralization activity (Figure 6F), such as alkaline phosphatase. In addition, we found increased expression of *TFIP11*, a ubiquitously expressed gene that suppresses chondrocytic development, may function during the transition from a cartilage template towards the formation of ossification centers [21] and may regulate tooth enamel deposition [22]. These *in vitro* results suggest that the ACVR1 R206H mutation confers increased propensity towards mineral deposition, possibly *via* a mineralized cartilage mechanism with transient increases in osteogenic markers.

## Discussion

**Figure 7:**
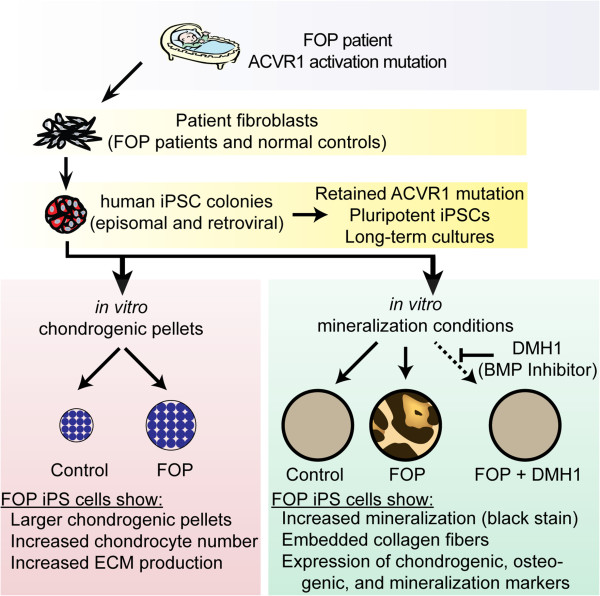
**Human iPS cells for disease modeling.** Our results suggest an increased disposition towards mineralization and chondrogenesis, consistent with modeling the early developmental and heterotopic bone formation processes. Our *in vitro* model will allow detailed interrogation of the mechanisms regulating bone formation and triggers of FOP flares, potentially identifying novel treatments for treating pathologic bone diseases.

Here, we use human iPS cells derived from FOP donors to establish a cellular foundation to understand the mechanisms underlying this dramatic disease (Figure 7). Our FOP iPS cells are a valuable *in vitro* human model system for understanding how human bone develops and identifying what control points may be amenable for manipulating normal and pathologic bone formation. The iPS cells will also facilitate identifying the triggers of heterotopic bone formation and strategies to block the different steps of mineralization or ossification, particularly by providing the ability to create key human cell types not directly available from the FOP patients or from patient progenitor cells. These iPS cells also provide a unique human-specific perspective that complements the *in vivo* studies of the FOP mouse model [23]. In this study, we created and analyzed three sets of independently-derived iPS cell lines. Our results suggest that generating iPS cells with integration-free methods rather than retroviral methods may decrease the likelihood of confounded results from persistent transgene expression, as has been described for *C-MYC*[24].

Our findings indicate that the ACVR1 R206H mutation can allow human iPS cells to form chondrocytes and mineralize *in vitro* without a clear fibro-proliferative stage as in FOP patients. Since the fibro-proliferative cells in an early FOP lesion are likely a collection of diverse cell types, we speculate that more primitive cell types or early skeletal precursors, such as those potentially in our iPS cell model, could be major contributors to the early FOP lesion. Although this might explain the mineralization that occurred without altered osteogenic markers, further experiments are needed to delineate the distinct effects of ACVR1 R206H on mineralization and osteogenesis in our iPS cell model.

Our results also suggest that the ACVR1 R206H mutation may have greater influence earlier in endochondral bone formation (i.e., at the chondrogenesis stage) based on gene expression patterns. Although *ACVR1* can be regulated by miR-148a [25], the decreasing levels of *ACVR1* R206H mRNA during mineralizing culture suggest that the 617 G > A mutation confers allele-specific regulation of RNA transcripts. However, the heterogeneity of our iPS cell mineralization cultures precluded us from identifying if a specific cell type is down-regulating ACVR1 R206H expression during culture. Delineating this potential regulatory function using FACS-purified cells or iPS cell-derived cell types could identify more specific roles for ACVR1 in bone formation and identify a new therapeutic target that would complement strategies to modulate the signaling properties of the ACVR1 receptor.

Although we could identify differences in mineralization by the FOP iPS cells, our simple culture conditions may not fully recapitulate the requirements for forming mature osteogenic cells from iPS cells. Similar challenges for creating mature cell types have been identified using current culture methods in other types of ES or iPS cell-derived progeny [26] and in some mesenchymal stem cell culture conditions [27]. This could account for the low expression levels of osteogenic genes. Instead, our culture conditions may better reflect the early steps of matrix mineralization in injured cartilage. The increased expression of genes associated with mineralization activity, such as *TFIP11* and alkaline phosphatase, suggest the ACVR1 R206H mutation can direct mineral deposition in tissues. This is consistent with reports indicating that BMP signaling increases mineralization activity in other cell types [28]. Bone formation and mineralization can also occur in the absence of osteoblasts in Osx-deficient mice [29,30].

Future studies will benefit from newer 3D scaffolds, the creation of multi-cellular bone models with iPS cell–derived cell types, better defined culture conditions, and *in vivo* translational assays which may favor the formation of mature skeletal cells. These directions will help identify the factors that may act at different stages of osteogenesis, including those that initiate heterotopic bone formation and lead to the formation of mature skeletal elements in humans. In addition, our results suggest that blocking mineralization induced by ACVR1 R206H may be a useful treatment strategy for preventing full mineralization of heterotopic bone lesions in FOP patients, thus preserving some limited joint mobility even if cartilage formation still occurs.

The increased chondrogenesis and mineralization, as well as the trend towards increased expression of endothelial cell gene markers, raise the possibility that the R206H ACVR1 mutation promotes skeletogenesis by affecting cell fate. Several observations support this notion. Studies in connective tissue progenitor cells from discarded primary teeth (SHED cells) of FOP patients with the ACVR1 R206H mutation show enhanced osteogenic differentiation [31]. In addition, introducing the R206H ACVR1 mutation into endothelial cells can increase endothelial-mesencymal transition and promote entry into an osteogenic lineage [32], possibly contributing to the bone formation in FOP. Finally, BMP activation induces differentiation in human ES cells [15]. These results suggest that activating the BMP signaling pathway may affect a cell’s ability to retain cell-fate commitment under specific circumstances.

Our studies showed that pluripotent FOP iPS cells can be generated without a BMP inhibitor using either the retrovirus or episomal methods. Prior attempts to make iPS cells from FOP fibroblasts by the Sendai virus method showed cellular instability [33]. This could be partially mitigated by culturing the cells with a BMP inhibitor, making the iPS cell formation process a useful assay for identifying small molecules that affect ACVR1 signaling. Although our study was not designed to directly compare the different iPS cell-generating methods, our results identify two major implications. First, our findings suggest that different pluripotency induction techniques, culture conditions, or embryonic fibroblast feeder cells could influence the success of iPS cell generation. Although Sendi virus can efficiently express reprogramming factors, the steps needed to remove replicating virus from cultures and the sensitivity of the viral RNA replicase to transgene sequences may contribute to different overall efficiencies of iPS cell generation [34]. Furthermore, the conditions under which iPS cells are derived, including the specific feeder cells used, can affect important aspects such as gene expression, reprogramming efficiency, and X-inactivation [35]. These observations indicate that the specific iPS cell derivation conditions may significantly affect the ability to generate iPS cell lines particularly if a disease mutation affects cell fate stability. Second, our study applies the FOP iPS cells as a disease model to demonstrate a potential function for ACVR1 in chondrogenesis and mineralization. These two endpoints may be useful for screening drug modulators that target different stages of human osteogenesis, particularly once the chondrogenic potential of the episomal iPS cells has been determined. Further studies to identify if cell fate stability is affected in non-pluripotent cells such as chondrocytes or osteoblasts may also reveal if cell fate plays a key role in the pathogenesis of heterotopic ossification in FOP.

## Conclusions

Our iPS cell strategy provides a key first step towards dissecting the cellular and molecular mechanisms of human skeletal disease pathogenesis in FOP. Human iPS cells will also be a valuable tool that reflects the diversity of patients we see in the clinic since patients, and thus, their iPS cells show clinical variability, genetic background effects, and epigenetic influences. Creation of new isogenic controls by gene correction [36], combined with translational genetic studies and correlations with murine models, provides exciting venues for new strategies to understand the stages of normal and pathologic human skeletal development. Our studies demonstrate the creation of iPS cells from patients with FOP, identify that ACVR1 may have a new role in regulating mineralization activity, and provide a proof-of-concept for further development of human iPS cells as disease models for studying human skeletal diseases.

## Abbreviations

BMP: Bone morphogenetic protein; EB: Embryoid body; ES cells: Embryonic stem cells; FOP: Fibrodysplasia ossificans progressiva; HDF: Human dermal fibroblast; iPS cells: Induced pluripotent stem cells; MSC: Mesenchymal stem cells.

## Competing interests

The authors have no competing interests to declare.

## Authors’ contributions

ECH, JT, TO, SY, and BC conceived and designed the experiments. ECH, YM, YH, CRS, HK, TDN, SS, SB, EB, AN, IA, and MI derived the iPS cell lines and performed the experiments. ECH and MI prepared the manuscript. All authors read and approved the final manuscript.

## Supplementary Material

Additional file 1: Table S1 Characteristics of the iPS cells used in this study. This table summarizes the characterization results for the FOP and control iPS cell lines used in this study and the original fibroblast source numbers for the iPS cell lines. Click here for file

Additional file 2: Figure S1 FOP dermal fibroblasts do not spontaneously mineralize. **(A)** Human dermal fibroblasts (HDFs) from patients with FOP are heterozygous for the *ACVR1* R206H (617G > A) mutation. **(B)** Primary human mesenchymal stem cells (MSCs) cultured in mineralizing media for 12 days show an increase in von Kossa staining. Mineralized deposits appear as the darker brown or black staining. The light golden staining is background. Standard MSC maintenance medium is used as a comparison for non-mineralizing conditions. **(C)** HDFs from FOP donors do not mineralize (black staining) when cultured in mineralizing medium for 18 days. Standard HDF medium without osteogenic supplements is used as a comparison for non-mineralizing conditions. Click here for file

Additional file 3: Table S2 Quantitative and RT-PCR primers used in this study. Click here for file

Additional file 4: Figure S2 Characterization of retroviral FOP iPS cells. **(A)** Teratoma formation showing representatives of the three germ layers. Scale bars = 200 μm. **(B)** Quantitative PCR gene expression analysis showing expression of pluripotency markers by the iPS cells. Error bars are average expression +/− 1 SD of technical triplicates. Gene expression studies were repeated in triplicate. Click here for file

Additional file 5: Figure S3 Characterization of additional retroviral FOP iPS cells. **(A)** Relative expression of retroviral transgenes in vFOP4-1, vFOP4-3, and vFOP5-22 iPS cells analyzed by quantitative RT-PCR. The value of each transgene 6 days after infection of wild-type dermal fibroblast (DF 4 F D6) was set to 1, demonstrating suppression of transgene expression. **(B)** Teratoma formation showing representatives of the three germ layers. Scale bars = 200 μm. **(C)** Karyotypes of the vFOP4 and vFOP5 iPSC lines are normal. **(D)** RT-PCR gene expression analysis showing expression of pluripotency markers by the iPS cells. **(E)** Quantitative PCR gene expression analysis showing expression of *RUNX2*, *SP7/OSX*, and *OC* genes during iPS cell mineralization culture. Error bars are average expression +/− 1 SD of measurements pooled from vFOP4-1, vFOP4-3, and vFOP5-22 iPS cell lines. n = 3 per time point. *, p < 0.05. Click here for file

Additional file 6: Figure S4 Characterization of episomal iPS cell lines. **(A)** FOP human dermal fibroblasts and the derived eFOP iPS cells have normal karyotypes. **(B)** Teratoma formation showing representatives of the three germ layers. b, bone; c, cartilage; g, primitive gut; m, melanocytes; n, neuronal tube like structures. Line eFOP2-8 showed no identifiable endodermal structures, and so was excluded from further analysis. Black scale bar = 50 μm. **(C)** Quantitative RT-PCR analysis of episomal transgene expression in the integration-free iPS cell lines. HDFs electroporated with the episomal vectors are used to determine the control levels of total mRNA present. Log_10_ scale. **(D)** Quantitative PCR analysis of the *EBNA* gene in genomic DNA from the integration-free iPS cell lines. Note that although episomal transgene expression of *OCT4* was detectable in the eFOP3-4 cell line, the level is low and no EBNA integration into the genome was detected. Click here for file

Additional file 7: Figure S5 Immunohistochemistry of episomal iPS cell colonies show expression of pluripotency markers NANOG, OCT3/4, SSEA3, TRA1-60, and TRA1-81. Click here for file
