# Supplementary figures and images for: Induced pluripotent stem cells from patients with human fibrodysplasia ossificans progressiva show increased mineralization and cartilage formation
Source: Orphanet J Rare Dis. 2013 Dec 9;8:190. doi: 10.1186/1750-1172-8-190 (PMC3892046; doi:10.1186/1750-1172-8-190)

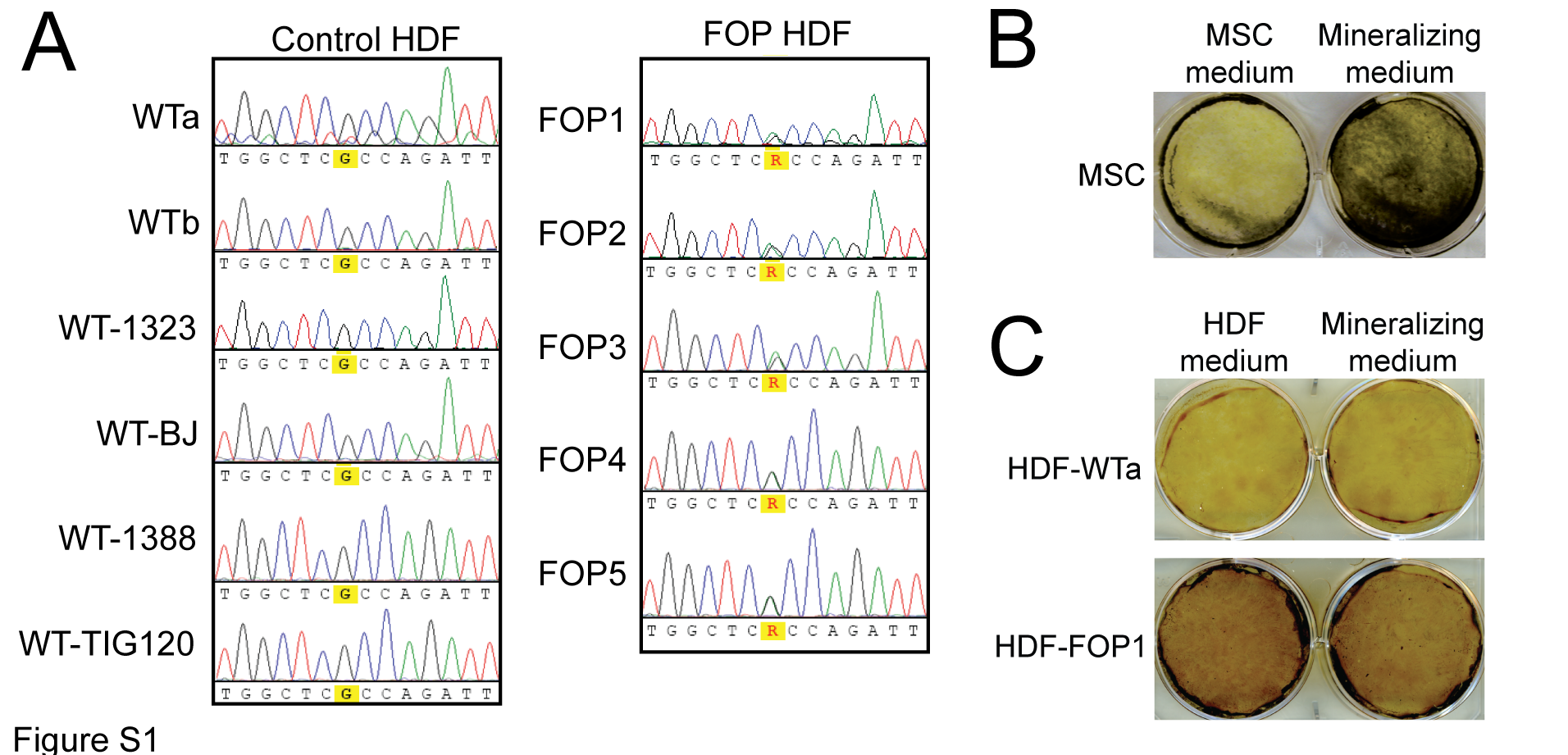

Supplement: Additional file 2: Figure S1 — FOP dermal fibroblasts do not spontaneously mineralize. (A) Human dermal fibroblasts (HDFs) from patients with FOP are heterozygous for the ACVR1 R206H (617G > A) mutation. (B) Primary human mesenchymal stem cells (MSCs) cultured in mineralizing media for 12 days show an increase in von Kossa staining. Mineralized deposits appear as the darker brown or black staining. The light golden staining is background. Standard MSC maintenance medium is used as a comparison for non-mineralizing conditions. (C) HDFs from FOP donors do not mineralize (black staining) when cultured in mineralizing medium for 18 days. Standard HDF medium without osteogenic supplements is used as a comparison for non-mineralizing conditions. [file 1750-1172-8-190-S2.tiff]

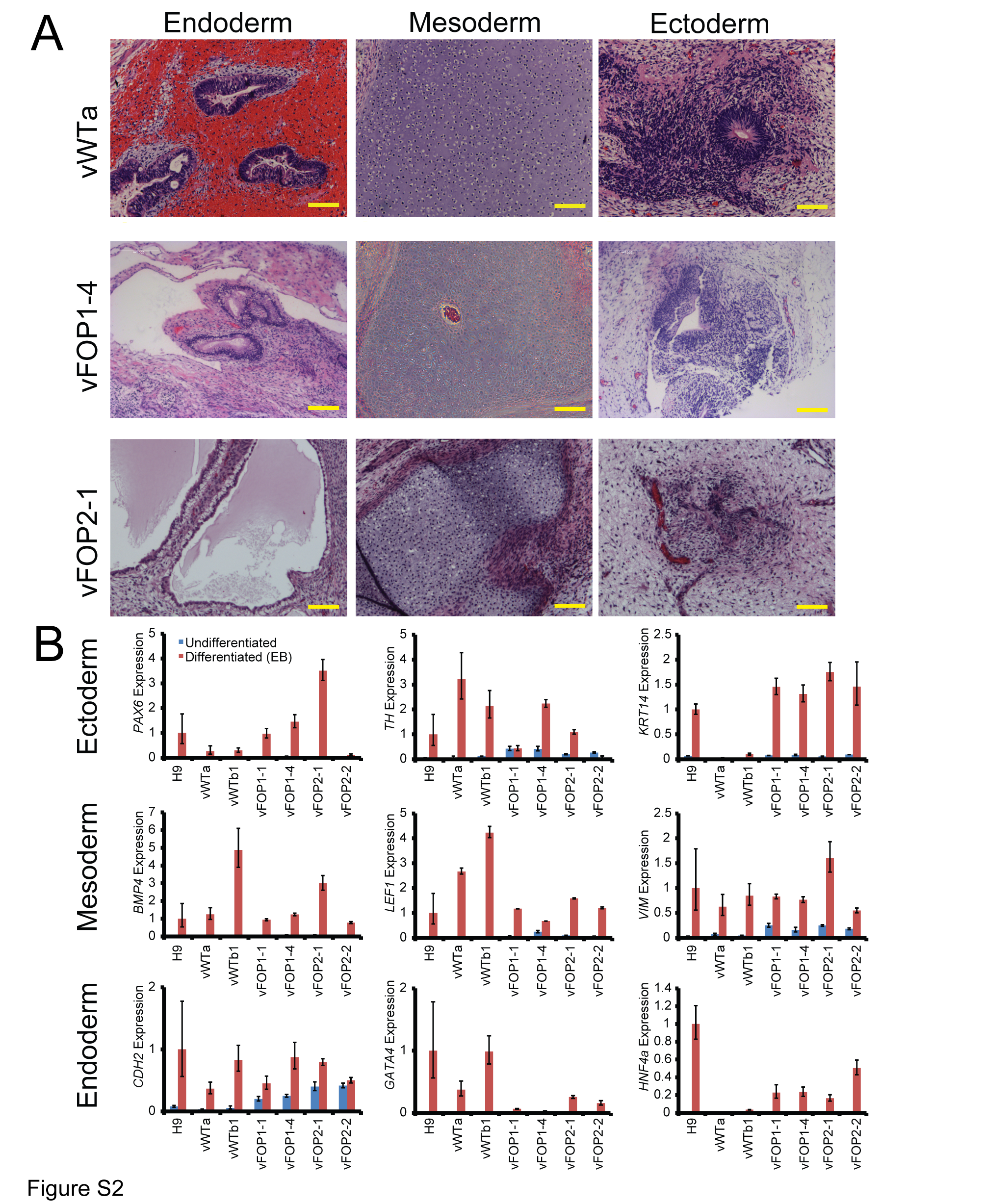

Supplement: Additional file 4: Figure S2 — Characterization of retroviral FOP iPS cells. (A) Teratoma formation showing representatives of the three germ layers. Scale bars = 200 μm. (B) Quantitative PCR gene expression analysis showing expression of pluripotency markers by the iPS cells. Error bars are average expression +/− 1 SD of technical triplicates. Gene expression studies were repeated in triplicate. [file 1750-1172-8-190-S4.tiff]

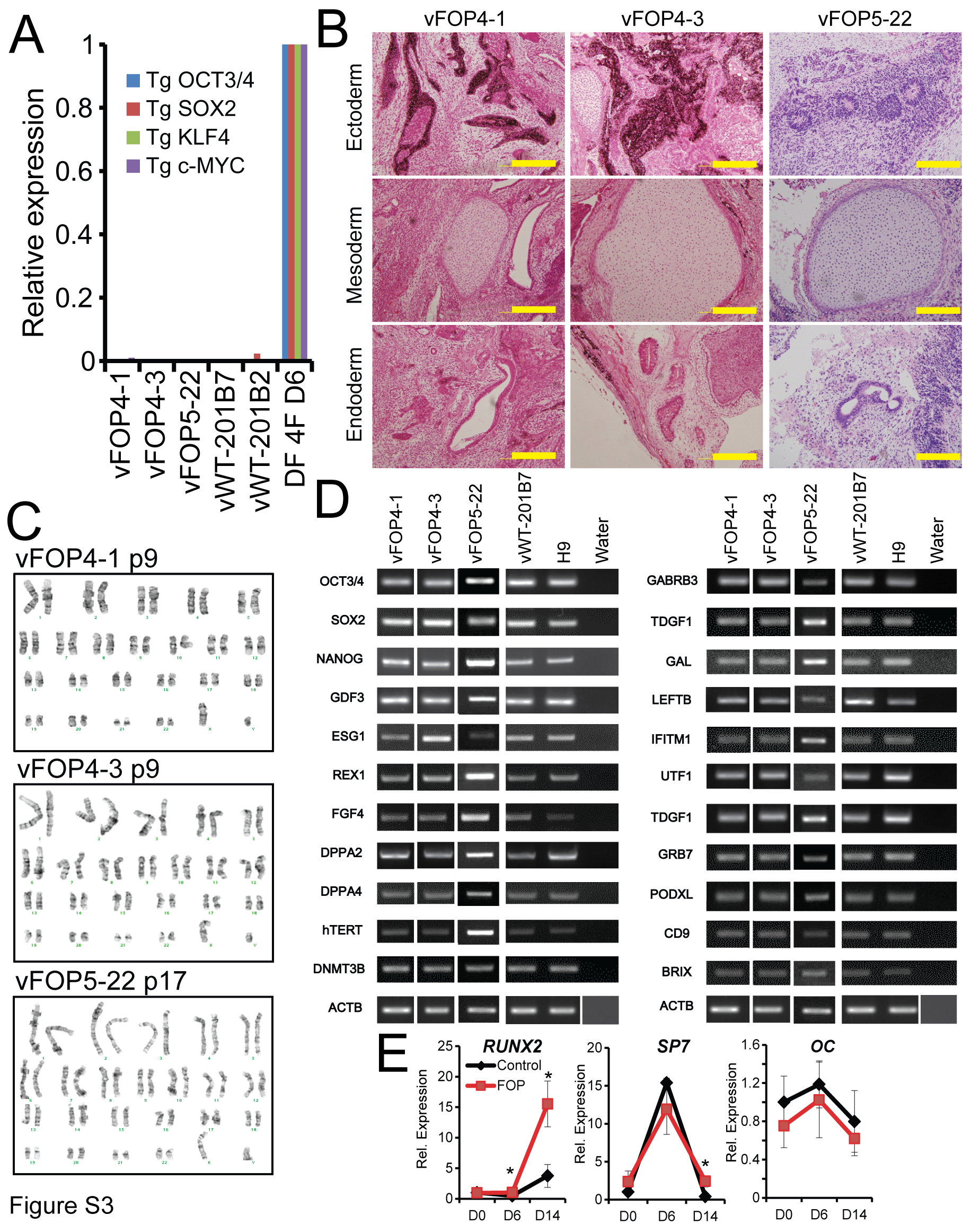

Supplement: Additional file 5: Figure S3 — Characterization of additional retroviral FOP iPS cells. (A) Relative expression of retroviral transgenes in vFOP4-1, vFOP4-3, and vFOP5-22 iPS cells analyzed by quantitative RT-PCR. The value of each transgene 6 days after infection of wild-type dermal fibroblast (DF 4 F D6) was set to 1, demonstrating suppression of transgene expression. (B) Teratoma formation showing representatives of the three germ layers. Scale bars = 200 μm. (C) Karyotypes of the vFOP4 and vFOP5 iPSC lines are normal. (D) RT-PCR gene expression analysis showing expression of pluripotency markers by the iPS cells. (E) Quantitative PCR gene expression analysis showing expression of RUNX2, SP7/OSX, and OC genes during iPS cell mineralization culture. Error bars are average expression +/− 1 SD of measurements pooled from vFOP4-1, vFOP4-3, and vFOP5-22 iPS cell lines. n = 3 per time point. *, p < 0.05. [file 1750-1172-8-190-S5.tiff]

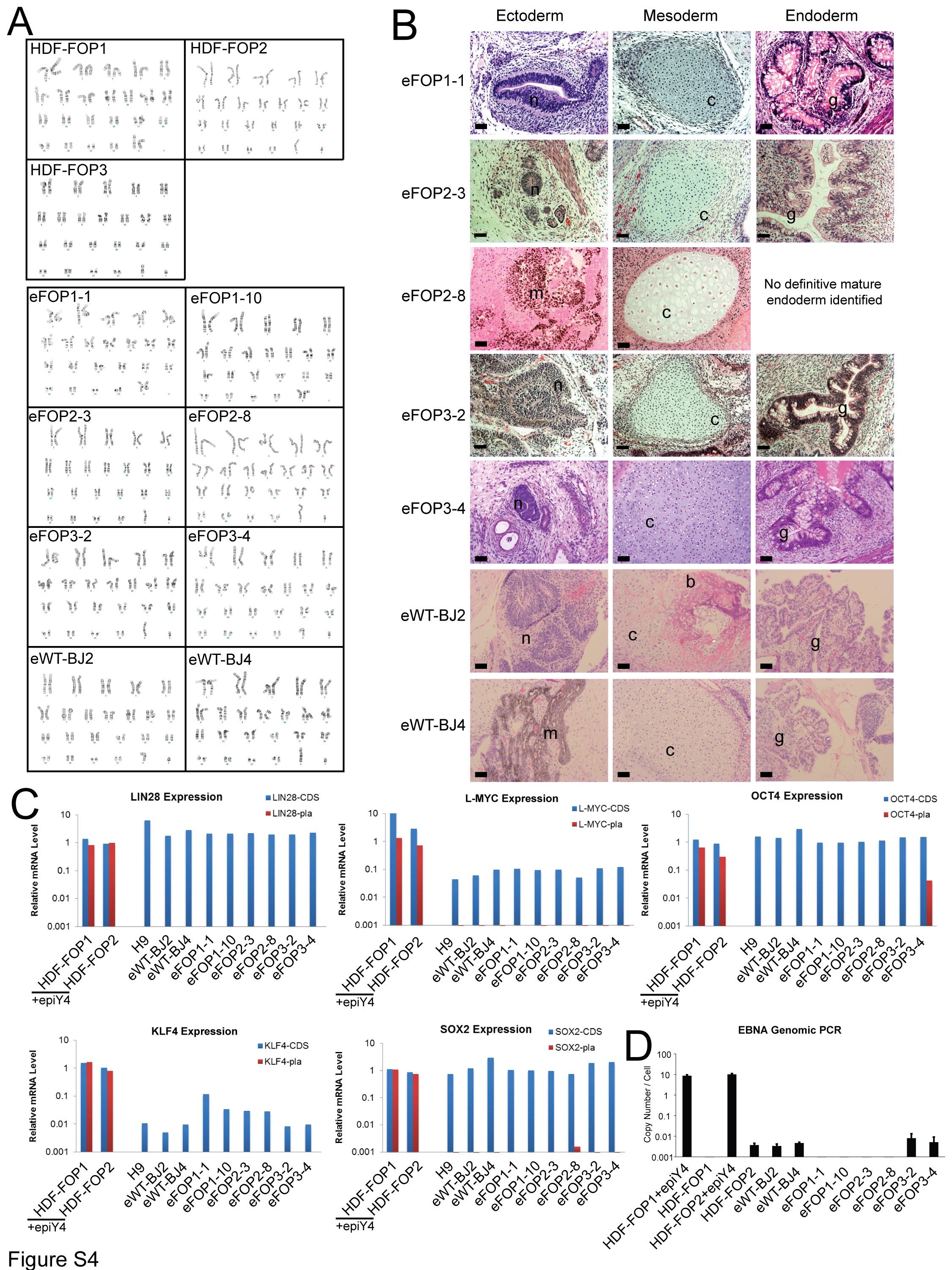

Supplement: Additional file 6: Figure S4 — Characterization of episomal iPS cell lines. (A) FOP human dermal fibroblasts and the derived eFOP iPS cells have normal karyotypes. (B) Teratoma formation showing representatives of the three germ layers. b, bone; c, cartilage; g, primitive gut; m, melanocytes; n, neuronal tube like structures. Line eFOP2-8 showed no identifiable endodermal structures, and so was excluded from further analysis. Black scale bar = 50 μm. (C) Quantitative RT-PCR analysis of episomal transgene expression in the integration-free iPS cell lines. HDFs electroporated with the episomal vectors are used to determine the control levels of total mRNA present. Log10 scale. (D) Quantitative PCR analysis of the EBNA gene in genomic DNA from the integration-free iPS cell lines. Note that although episomal transgene expression of OCT4 was detectable in the eFOP3-4 cell line, the level is low and no EBNA integration into the genome was detected. [file 1750-1172-8-190-S6.tiff]

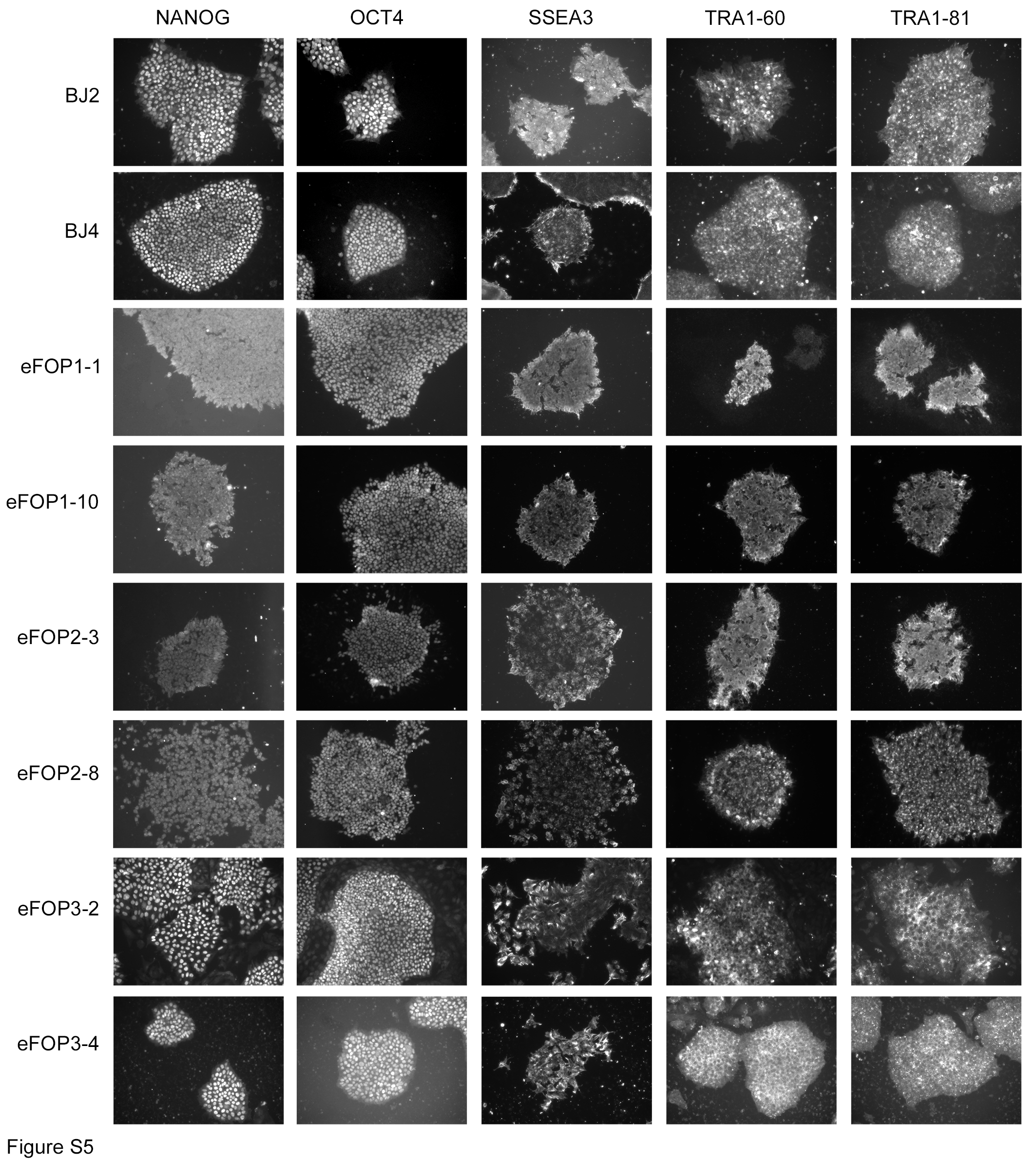

Supplement: Additional file 7: Figure S5 — Immunohistochemistry of episomal iPS cell colonies show expression of pluripotency markers NANOG, OCT3/4, SSEA3, TRA1-60, and TRA1-81. [file 1750-1172-8-190-S7.tiff]
