# Supplementary material for: Induced pluripotent stem cells from patients with human fibrodysplasia ossificans progressiva show increased mineralization and cartilage formation
Source: Orphanet J Rare Dis. 2013 Dec 9;8:190. doi: 10.1186/1750-1172-8-190 (PMC3892046; doi:10.1186/1750-1172-8-190)
Supplement: Additional file 3: Table S2 — Quantitative and RT-PCR primers used in this study. [file 1750-1172-8-190-S3.docx]

**Supplemental Table S2: Primers**

| **Taqman Primers – gene expression** | |
| --- | --- |
| **Sequence** | **Taqman probe ID** |
| ALPL | Hs01029144_m1 |
| ACAN | Hs00153936_m1 |
| ACVR1 | Hs00153836_m1 |
| BGLAP / OC | Hs00609452_g1 |
| COL2a1 | Hs01060334_g1 |
| COMP | Hs00164359_m1 |
| DLX5 | Hs00193291_m1 |
| GAPDH | Hs99999905_m1 |
| ID1 | Hs03676575_s1 |
| NANOG | Hs02387400_g1 |
| RUNX2 | Hs00231692_m1 |
| SOX9 | Hs00165814_m1 |
| SP7 / OSX | Hs005417229_m1 |
| TERT | Hs00972656_m1 |
| TFIP11 | Hs00201749_m1 |
| Total OCT4 | Hs00999632_g1 |
| Total SOX2 | Hs01053049_s1 |
| Total KLF4 | Hs01034973_g1 |
| Total MYCL1 | Hs00420495_m1 |
| Total LIN28A | Hs00702808_s1 |

| **Sybr Green primers – gene expression** | | |
| --- | --- | --- |
|  | Forward Primer | Reverse Primer |
| hACVR1 WT | TGGTACAAAGAACAGTGGCTAG | CCATACCTGCCTTTCCCGA |
| hACVR1 R206H | TGGTACAAAGAACAGTGGCTTA | CCATACCTGCCTTTCCCGA |
| hGAPDH | AGATCATCAGCAATGCCTCCTG | ATGGCATGGACTGTGGTCATG |

References: J Kaplan et al, Gene Ther, 2011; Liori, et al., JBMR, 2006

| **Sybr Green primers for gene expression analysis** | | |
| --- | --- | --- |
|  | Forward Primer | Reverse Primer |
| hSOX9 | GACTTCCGCGACGTGGAC | GTTGGGCGGCAGGTACTG |
| hCOL2A1 | GGCAATAGCAGGTTCACGTACA | CGATAACAGTCTTGCCCCACTT |
| hCOMP | CAACTGTCCCCAGAAGAGCAA | TGGTAGCCAAAGATGAAGCCC |

| **Retroviral iPS cell gene expression (RT-PCR; Takahashi, et al. 2007)** | | |
| --- | --- | --- |
|  | Forward Primer | Reverse Primer |
| hOCT3/4 | S944:  CCCCAGGGCCCCATTTTG GTA CC | MXs-L3205: CCCTTTTTCTGGAGACTAAATAAA |
| hSOX2 | S691: GGCACCCCTGGCATGGCTCTTGGCTC | pMXs-L3205: CCCTTTTTCTGGAGACTAAATAAA |
| hKLF4 | S1128:  ACGATCGTGGCCCCGGAAAAGGACC | pMXs-L3205: CCCTTTTTCTGGAGACTAAATAAA |
| hcMYC | S1011:  CAACAACCGAAAATGCACCAGCCCCAG | pMXs-L3205: CCCTTTTTCTGGAGACTAAATAAA |
| hACTB | CTATCCCTGTACGCCTCTGG | CCATCTCTTGCTCGAGTCC |

| **Episomal plasmid iPS cell Sybr green primers (Okita, et al. 2011)** | | |
| --- | --- | --- |
|  | Forward Primer | Reverse Primer |
| Pla-OCT4 | CATTCAAACTGAGGTAAGGG | TAGCGTAAAAGGAGCAACATAG |
| Pla-SOX2 | TTCACATGTCCCAGCACTACCAGA | TTTGTTTGACAGGAGCGACAAT |
| Pla-KLF4 | CCACCTCGCCTTACACATGAAGA | TAGCGTAAAAGGAGCAACATAG |
| Pla-MYCL1 | GGCTGAGAAGAGGATGGCTAC | TTTGTTTGACAGGAGCGACAAT |
| Pla-LIN28 | AGCCATATGGTAGCCTCATGTCCGC | TAGCGTAAAAGGAGCAACATAG |
| EBNA-1 | ATCAGGGCCAAGACATAGAGATG | GCCAATGCAACTTGGACGTT |
